# Randomised Phase 2 study of lapatinib and vinorelbine vs vinorelbine in patients with HER2 + metastatic breast cancer after lapatinib and trastuzumab treatment (KCSG BR11-16)

**DOI:** 10.1038/s41416-019-0618-z

**Published:** 2019-11-06

**Authors:** Sung Hoon Sim, In Hae Park, Kyung Hae Jung, Sung-Bae Kim, Jin-Hee Ahn, Kyung-Hun Lee, Seock-Ah Im, Young-Hyuck Im, Yeon Hee Park, Joohyuk Sohn, Yu Jung Kim, Suee Lee, Hee-Jun Kim, Yee Soo Chae, Kyong Hwa Park, Byung-Ho Nam, Keun Seok Lee, Jungsil Ro

**Affiliations:** 1 Center for Breast Cancer, Research Institute, National Cancer Center, Goyang, Korea; 2 Asan Medical Center, University of Ulsan, College of Medicine, Seoul, Korea; 3 Seoul National University Hospital, Cancer Research Institute, Seoul National University, College of Medicine, Seoul, Korea; 4 Samsung Medical Center, Sungkyunkwan University, School of Medicine, Seoul, Korea; 5 Yonsei University, College of Medicine, Yonsei Cancer Center, Seoul, Korea; 6 Division of Hematology and Medical Oncology, Department of Internal medicine, Seoul National University Bundang Hospital, Bundang, Korea; 7 Department of Internal medicine, Dong-A University, College of Medicine, Busan, Korea; 8 Chung-Ang University, College of Medicine, Seoul, Korea; 9 Kyungpook National University, College of Medicine, Daegu, Korea; 10 Department of Internal Medicine, Division of Oncology/Hematology, Korea University, Seoul, Korea; 11 Biometric Research Branch, Division of Cancer Epidemiology and Prevention, Research Institute & Hospital, National Cancer Center, Goyang, Korea

**Keywords:** Breast cancer, Breast cancer

## Abstract

**Background:**

The continuum of anti-HER2 agents is a standard treatment of HER2 + metastatic breast cancer (MBC). This study evaluated the efficacy of lapatinib plus vinorelbine in patients progressed on both trastuzumab and lapatinib treatments.

**Methods:**

A total of 149 patients were randomly assigned to lapatinib with vinorelbine (LV) (*n* = 75; lapatinib, 1000 mg daily; vinorelbine 20 mg/m^2^ D1, D8 q3w) or vinorelbine (V) (*n* = 74; 30 mg/m^2^ D1, D8 q3w). The primary endpoint was progression-free survival (PFS) rate at 18 weeks.

**Results:**

The median number of previous anti-HER2 therapies was 2 (range 2–5). There was no significant difference in PFS rate at 18 weeks between LV and V arms (45.9% vs 38.9%, *p* = 0.40). ORR was 19.7% in LV arm, and 16.9% in V arm (*p* = 0.88). PFS and OS did not differ between two arms (LV vs V; median PFS, 16 vs 12 weeks, HR = 0.86, 95% CI 0.61–1.22; median OS, 15.0 vs 18.9 months, HR = 1.07, 95% CI 0.72–1.58). Toxicity profiles were similar in both arms and all were manageable.

**Conclusions:**

Lapatinib plus vinorelbine treatment was tolerable; however, it failed to demonstrate the clinical benefits over vinorelbine alone in patients with HER2 + MBC after progression on both trastuzumab and lapatinib.

**Clinical trial registration:**

ClinicalTrials.gov number NCT01730677.

## Background

Recent advances in human epidermal growth factor receptor 2 (HER2) therapy have led to new paths of treatment in HER2-positive advanced breast cancer. Since the first anti-HER2 monoclonal antibody, trastuzumab, was introduced into the treatment of HER2-positive breast cancer, new anti-HER2 agents have been developed and incorporated into the armamentarium over two decades. As a result, the median overall survival (OS) of metastatic HER2-positive breast cancer patients has reached 56 months.^1^ Despite effective new agents, metastatic breast cancer remains incurable since the tumours eventually acquire resistance to the agents. Therefore, it is still necessary and challenging to develop subsequent treatment strategies.

Previous clinical trials of the combination of lapatinib or trastuzumab after trastuzumab failure yielded better survival than treatment with chemotherapy alone.^2–4^ Therefore, maintaining anti-HER2 treatment has been strongly recommended and becomes the mainstay,^5^ even after treatments in combination with anti-HER2 therapy show failure.

Lapatinib is a small molecule that inhibits epidermal growth factor receptor/HER2 signalling in cancer cells, and its treatment efficacy has been well explored in breast cancer.^2,4,6^ However, the efficacy of continuing lapatinib after the failure of both trastuzumab and lapatinib has not been evaluated. This study aimed to evaluate the efficacy of adding lapatinib to vinorelbine in patients who experienced disease progression after lapatinib and trastuzumab treatment.

## Patients and methods

### Study design and patients

This open-label, multicentre, randomised phase 2 study was conducted by Korean Cancer Study Group (KCSG, KCSG BR11-16) in South Korea. Patients with metastatic HER2-positive breast cancer were eligible if (1) they were 20 years and older with an Eastern Cooperative Oncology Group performance status score of 0–1 or 2; (2) they had previously received anthracycline-based chemotherapy; (3) the disease had progressed on lapatinib treatment with the best response of complete/partial response (CR/PR), or at least, stable disease (SD) for 12 weeks or more and (4) they previously received at least two palliative treatment regimens containing anti-HER2 agents (T-DM1, trastuzumab, pertuzumab or lapatinib). Other eligibility criteria were the resolution of therapy-related toxicities of grade 1 or lower according to National Cancer Institute Common Terminology Criteria for Adverse Events version 4.0 with adequate organ function and a negative pregnancy test. HER2 positivity was defined as 3 + on immunohistochemical staining or 2 + with positive fluorescence in situ hybridisation (silver in situ hybridisation or chromogenic in situ hybridisation were accepted) according to the guideline of the American Society of Clinical Oncology and College of American Pathologists.^7^ The patients with symptomatic brain metastases, and serious medical problems such as heart failure, uncontrolled diabetes or uncontrolled infection were excluded.

The patients were randomised to either arm receiving a combination of lapatinib plus vinorelbine (LV) or vinorelbine alone (V) by computer-generated allocation. Randomisation was stratified according to previous response to lapatinib (CR + PR vs SD) and the presence of visceral metastasis. The primary endpoint of this study was the progression-free survival (PFS) rate at 18 weeks and the secondary endpoints included objective response rate (ORR), PFS safety profiles and OS.

### Study treatment and assessment

In the LV arm, patients received oral lapatinib, 1000 mg daily, and intravenous infusions of vinorelbine at a dose of 20 mg/m^2^ on days 1 and 8, every 3 weeks. The dose of combination treatment was determined based on the previous data and modified for safety concerns.^8^ In the V arm, patients received 30 mg/m^2^ of vinorelbine on days 1 and 8, every 3 weeks. Tumours were assessed by using the Response Evaluation Criteria in Solid Tumours version 1.1 at screening and every 6 weeks from the initiation of treatment. Adverse events were evaluated and recorded according to the National Cancer Institute Common Terminology Criteria for Adverse Events version 4.0 at the baseline and throughout treatment.

### Statistical analysis

Sample size was considered based on the progression-free (survival) rate as a binary outcome at 18 weeks from treatment initiation as a primary endpoint. This primary endpoint was chosen to avoid possible biases in determining progression times in a non-blinded randomised trial like our study.^9^ We set the median progression-free survival to be 2.5 months for vinorelbine arm, and 4 months for lapatinib and vinorelbine combination arm based on previous studies.^10,11^ In this condition, the PFS rate at 18 weeks would be 31.6% for control arm, and 48.7% for combination arm. With 80% power and one-sided type I error rate of 0.1, a total of 142 patients were required. Considering 5% dropout, the trial would need to recruit ~150 patients.

All randomised patients were included in the intention-to-treat (ITT) population, which was used for all survival analyses. The progression-free survival rates at 18 weeks were estimated based on Kaplan–Meier method, and the differences between two groups were compared by using Greenwood’s variance estimator. Safety was accessed in the safety population that had received at least one dose of study treatment. The ORR was evaluated amongst patients who had measurable disease and calculated as the proportion of patients with complete or partial tumour response. PFS and OS were estimated by the Kaplan–Meier method, and compared by using the log-rank test. Cox proportional hazards analysis was used to assess the experimental treatment effect against the control with variables expected to affect treatment response.

## Results

### Baseline characteristics and study treatment

**Fig. 1:**
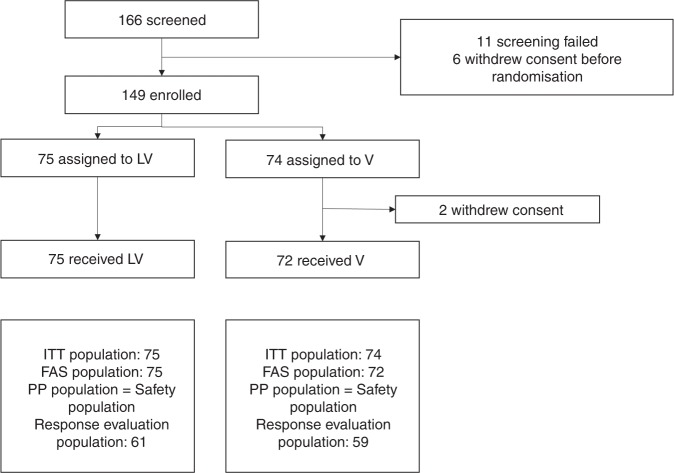
Study flowchart

Between December 2011 and March 2018, 166 patients were enrolled and followed up. Eleven patients showed screening failure, and six patients withdrew consent before randomisation. Therefore, 149 patients were randomised to the lapatinib plus vinorelbine arm (*n* = 75) and to the vinorelbine arm (*n* = 74) (Fig. 1). Two patients in the vinorelbine arm withdrew their consent after randomisation. Thus, 147 patients received at least one dose of the assigned treatment. There was no statistical difference in the baseline characteristics between the two groups (Table 1). The hormone receptor positivity rate was 53.4% amongst patients in the lapatinib plus vinorelbine arm and 45.9% in the vinorelbine arm.

**Table 1:** Patient characteristics

		Total *n* = 149	LV arm (*n* = 75)	V arm (*n* = 74)	*P-*value
Age, median	Median 53 (28–80)		54 (28–80)	52 (30–74)	0.349
ECOG PS*	0	38 (25.5%)	19	19	0.943
	1	108 (72.5%)	55	53	
	2	3 (2.0%)	1	2	
Menopausal status	Postmenopausal	117 (78.5%)	57	60	0.567
	Premenopausal	30 (20.1%)	17	13	
	Not applicable (hysterectomy)	2 (1.4%)	1	1	
De novo stage IV vs recurrence	De novo stage IV	51 (34.2%)	27	24	0.774
	Recurrence	98 (65.8%)	48	50	
Histology	IDC	147 (98.6%)	74	73	0.999
	ILC	1 (0.7%)	1	0	
	Others	1 (0.7%)	0	1	
ER/PgR	Positive	73 (49.0%)	39 (53.4)	34 (45.9)	0.565
	Negative	76 (51.0%)	36	40	
Visceral metastasis	Yes	75 (50.3%)	38	37	0.999
	No	74 (49.7%)	37	37	
Number of previous chemotherapy	1	18 (12.1%)	9	9	0.252
	2	59 (39.6%)	25	34	
	≧3	72 (48.3%)	41	31	
Previous lines of anti-HER2 treatment	2	121 (81.2%)	65	56	0.131
	≧3	28 (18.8%)	10	18	
Previous response to lapatinib	CR + PR	74 (49.6%)	38	36	0.934
	SD ≥ 12 weeks	75 (50.4%)	37	38	
Last lapatinib use	Within 6 months	103 (69.1)	52	51	0.999
	Before 6 months	46 (30.9)	23	23	

*ECOG PS* Eastern Cooperative Oncology Group performance status, *IDC* invasive ductal carcinoma, *ILC* invasive lobular carcinoma, *CR* complete response, *PR* partial response, *SD* stable disease

The number of previous anti-HER2 treatment regimens was 2 (range, 2–5). Seventy-four patients (49.6%) had CR or PR from prior lapatinib treatment, and 69.1% of patients had received the last dose of lapatinib within 6 months prior to randomisation.

### Efficacy

**Fig. 2:**
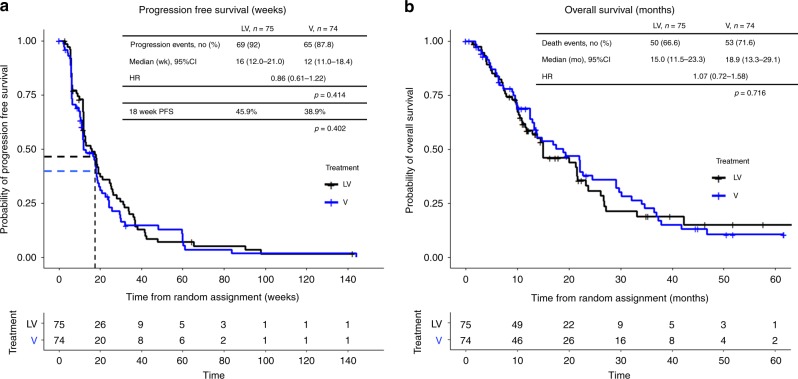
Survival analysis in the intention-to-treat (ITT) population. Kaplan–Meier curve for progression-free survival (PFS) (**a**) and overall survival (OS) (**b**) between lapatinib with vinorelbine (LV) and vinorelbine alone (V)

**Fig. 3:**
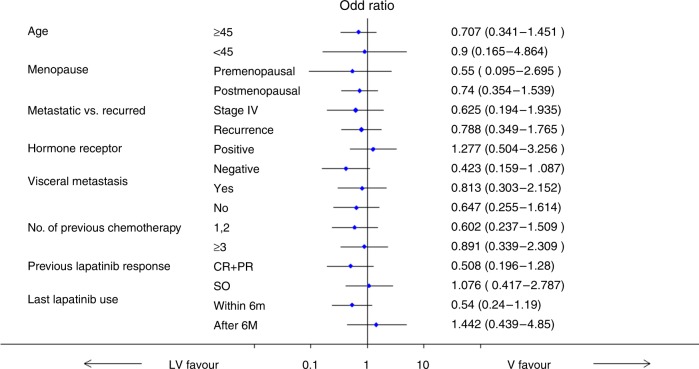
Subgroup analysis of 18-week progression-free survival (PFS). Hormone receptor negative, prior lapatinib response (CR or PR) and the last administration of lapatinib within 6 months favoured LV treatment but it did not show statistical significance

The 18-week PFS rate was 45.9% in the LV arm, and 38.9% in the V arm. The difference was 7.02% (95% CI, −9.4 to 23.43%), which was not significantly different (*P* = 0.402, Table 2). One hundred and twenty patients had measurable disease on the baseline images, which were evaluated for response analysis. Objective responses (CR + PR) were observed in 12 (19.7%) patients in LV arm (*n* = 61) and 10 (16.9%) in the V arm (*n* = 59), which showed no statistical difference (*P* = 0.881, Table 3). The median PFS was 16.0 weeks (95% confidence interval [CI] 12.0–21.0) in the LV arm and 12.0 weeks (95% CI 11.0–18.4) in the V arm (*P* = 0.414, Fig. 2a) without statistical difference. Likewise, the median OS was similar between the two arms (15.0 months, 95% CI 11.5–23.3 in the LV arm vs 18.9 months, 95% CI 13.3–29.1 in the V arm; *P* = 0.716, Fig. 2b). In the subgroup analysis for progression at 18 weeks, hormone receptor negativity, prior responsiveness to lapatinib (CR or PR) and the last dose of lapatinib within 6 months tended to favour LV combination treatment with no statistical significance (Fig. 3). In the subgroup analyses of PFS and OS, there was no difference between the two arms according to prior responsiveness to lapatinib and last lapatinib use (Supplementary Figs.).

**Table 2:** Progression-free survival rate at 18 weeks after treatment

	LV, *n* = 75	V, *n* = 74
18-week progression events, no. (%)	69 (92)	65 (87.8)
18-week PFS	45.9%	38.9%
		*p* = 0.402

**Table 3:** Response rate according to treatment arms

Objective response rate (ORR) (*n* = 120)		LV arm (*n* = 61)	V arm (*n* = 59)
	CR or PR	12	10
	SD/PD	49	49
ORR		19.67%	16.94%
			*P* = 0.8812
**FAS (*n*** **=** **147)**		**LV arm (*n*** **=** **75)**	**V arm (*n*** **=** **72)**
CBR	CR + PR + SD ≥ 24 weeks	25 (33.3%)	17 (23.6%)
	CR + PR + SD < 24 weeks or PD	50 (66.7%)	55 (76.4%)
			*P* = 0.192

*FAS* full analysis set, *CBR* clinical benefit ratio, *CR* complete response, *PR* partial response, *SD* stable disease

### Toxicities

The toxicities of both study drugs were mild and manageable, except for neutropenia. In the safety population, grade 3 or 4 neutropenia occurred at a rate of 44% in the LV arm and 65.2% in the V arm. The incidence of febrile neutropenia was low (6% in the LV arm and 8% in the V arm). No grade 3 or 4 skin toxicity or hand–foot syndrome was observed in the LV arm. Both arms showed comparable frequencies of the other forms of toxicity (Supplementary Table 1).

## Discussion

In this trial, we investigated the clinical benefit of adding lapatinib to vinorelbine in patients who experienced disease progression on both trastuzumab and lapatinib treatment. The combination treatment of lapatinib and vinorelbine was tolerable. However, clinical outcomes of the combination treatment were not different from those of vinorelbine alone.

After the failure of first-line anti-HER2 treatment, the continuum of anti-HER2 agents has become the mainstay of treatment.^5^ Currently, T-DM1 is recommended as the second-line therapy after trastuzumab plus pertuzumab-containing therapy. After that, trastuzumab plus other chemotherapeutic agents or lapatinib plus capecitabine is considered.^5,11,12^ At the time of beginning this study, T-DM1 or pertuzumab was not available yet at the routine practice in Korea. Therefore, the majority of patients in this study had been exposed only to trastuzumab and lapatinib before entering in this study.

Various resistance mechanisms of anti-HER2 therapy have been suggested, including upregulation of ligands, increased signalling from other HER family receptors and loss of phosphatase and tensin homologue (PTEN) with activation of the PI3K pathway.^13–15^ These mechanisms suggest that persistent HER2 signal activation can be one of the major drivers of tumour proliferation after trastuzumab failure. Recent report showed that ER signal is one of the HER2-resistance signal pathways, and blocking both ER and HER2 signal with fulvestrant and neratinib could be a good way to overcome HER2 resisatance.^16^ In this study, ~50% of patients had ER + /HER2 + breast cancer. However, unfortunately we could not evaluate the efficacy of prior endocrine treatment, which was not planned from the beginning.

In clinical trials pertaining to anti-HER2 treatment, lapatinib with capecitabine after trastuzumab failure exhibited longer PFS compared with capecitabine alone (combination therapy, 8.4 months vs monotherapy, 4.4 months).^2^ Combined lapatinib with trastuzumab treatment demonstrated prolonged survival compared with lapatinib as monotherapy.^4^ Later, trastuzumab in combination with capecitabine also showed better survival outcomes than capecitabine alone after trastuzumab failure.^3^

In the current trial, however, continuing lapatinib after progression while on two prior anti-HER2 agents failed to improve the PFS rate. According to our results, the reuse of lapatinib or continuing lapatinib after progression while on lapatinib did not seem to be clinically effective. This may be explained by the different mechanisms underlying trastuzumab resistance, which is associated with incomplete blockade of the HER2-related pathway.^17–19^ In contrast, lapatinib resistance could be related to the induction of other proliferation pathways such as hormone receptor signals.^17^ In our subgroup analysis, the hormone receptor-negative patients showed a tendency towards favourable outcome on lapatinib treatment compared with the hormone receptor-positive group. Such a different resistance mechanism may have affected the efficacy of lapatinib reuse.

On the other hand, HER2 receptors could be modified with the use of lapatinib and may affect the result, as suggested by some in vitro data where inactive HER2 receptors accumulated on tumour cells after lapatinib treatment.^20^ Although the accumulation is not linked directly to the resistance, it suggests that reuse of trastuzumab is a better alternative when the tumour has acquired resistance to lapatinib. This is also supported by a small outcome report. After progression on lapatinib treatment, retreatment with trastuzumab showed a 47% clinical benefit.^21^

Nowadays, the new generation of HER2-targeting tyrosine kinase inhibitors such as poziotinib and neratinib are also gaining attention. Poziotinib showed meaningful activity in heavily treated HER2-positive breast cancer cases^22^ and neratinib could be effective as third-line treatment compared with lapatinib (NALA trial, http://www.pumabiotechnology.com/pr20181217.html). They are irreversible pan-HER inhibitors and are expected to be more effective as anti-HER2 treatments than lapatinib, the reversible epidermal growth factor receptor/HER2 inhibitor.^23^

After progression on the next-generation HER2-targeted therapies, it is not known which agents are active. Despite the introduction of some agents that target HER2, there could be various clinical settings, in which the use of anti-HER2 agents is limited. To map out a good clinical strategy for patients with metastatic HER2-positive breast cancer, the investigation of the appropriate sequence of anti-HER2 agents will be needed in the future.

In conclusion, although the combination of lapatinib and vinorelbine was tolerable, it did not demonstrate added clinical benefits compared with single-agent chemotherapy vinorelbine in metastatic HER2-positive breast cancer patients when used beyond the second line after patient progression on trastuzumab and lapatinib.

## Supplementary information

Supplementary Table adn Ohter information

## Data Availability

Data supporting the results will be provided on public websites or archives.
