# Supplementary material for: Randomised Phase 2 study of lapatinib and vinorelbine vs vinorelbine in patients with HER2 + metastatic breast cancer after lapatinib and trastuzumab treatment (KCSG BR11-16)
Source: Br J Cancer. 2019 Nov 6;121(12):985–90. doi: 10.1038/s41416-019-0618-z (PMC6964682; doi:10.1038/s41416-019-0618-z)
Supplement: Supplementary file 1 — Supplementary Table adn Ohter information [file 41416_2019_618_MOESM1_ESM.docx]

Supplementary Table and other information

1. Supplementary Table1.
2. Supplementary Figure 1
3. Supplementary Figure 2
4. Supplementary legends
5. All institutional review board committees and approval No.

Supplementary Table1. Adverse events (AEs) in both arm

|  |  | **LV arm** | | | **V arm** | | |
| --- | --- | --- | --- | --- | --- | --- | --- |
|  |  | All grade | Gr3 | Gr4 | All grade | Gr3 | Gr4 |
| Hematologic AEs | Neutropenia | 39 | 15 | 18 | 49 | 25 | 20 |
|  | Febrile neutropenia | 5 | 3 | 2 | 6 | 3 | 2 |
|  | Anemia | 12 | 3 | 0 | 13 | 4 | 0 |
| Non-Hematologic AEs | Abdominal pain | 16 | 2 | 0 | 17 | 6 | 6 |
|  | Liver function abnormality | 6 | 0 | 0 | 15 | 3 | 0 |
|  | Anorexia | 15 | 1 | 0 | 18 | 0 | 0 |
|  | Enterocolitis | 2 | 2 | 0 | 1 | 0 | 0 |
|  | edema | 2 | 1 | 0 | 2 | 0 | 0 |
|  | Fatigue | 16 | 0 | 0 | 17 | 2 | 0 |
|  | Hypokalemia | 1 | 1 | 0 | 0 | 0 | 0 |
|  | Hypotension | 1 | 1 | 0 | 0 | 0 | 0 |
|  | Mucositis oral | 13 | 3 | 0 | 9 | 0 | 0 |
|  | Myalgia | 16 | 0 | 0 | 37 | 2 | 0 |
|  | Pneumonitis | 1 | 0 | 0 | 3 | 1 | 0 |
|  | Skin infection | 1 | 1 | 0 | 0 | 0 | 0 |
|  | Soft tissue infection | 1 | 1 | 0 | 0 | 0 | 0 |
|  | Urinary tract infection | 1 | 1 | 0 | 1 | 0 | 0 |

2. Supplement figure 1


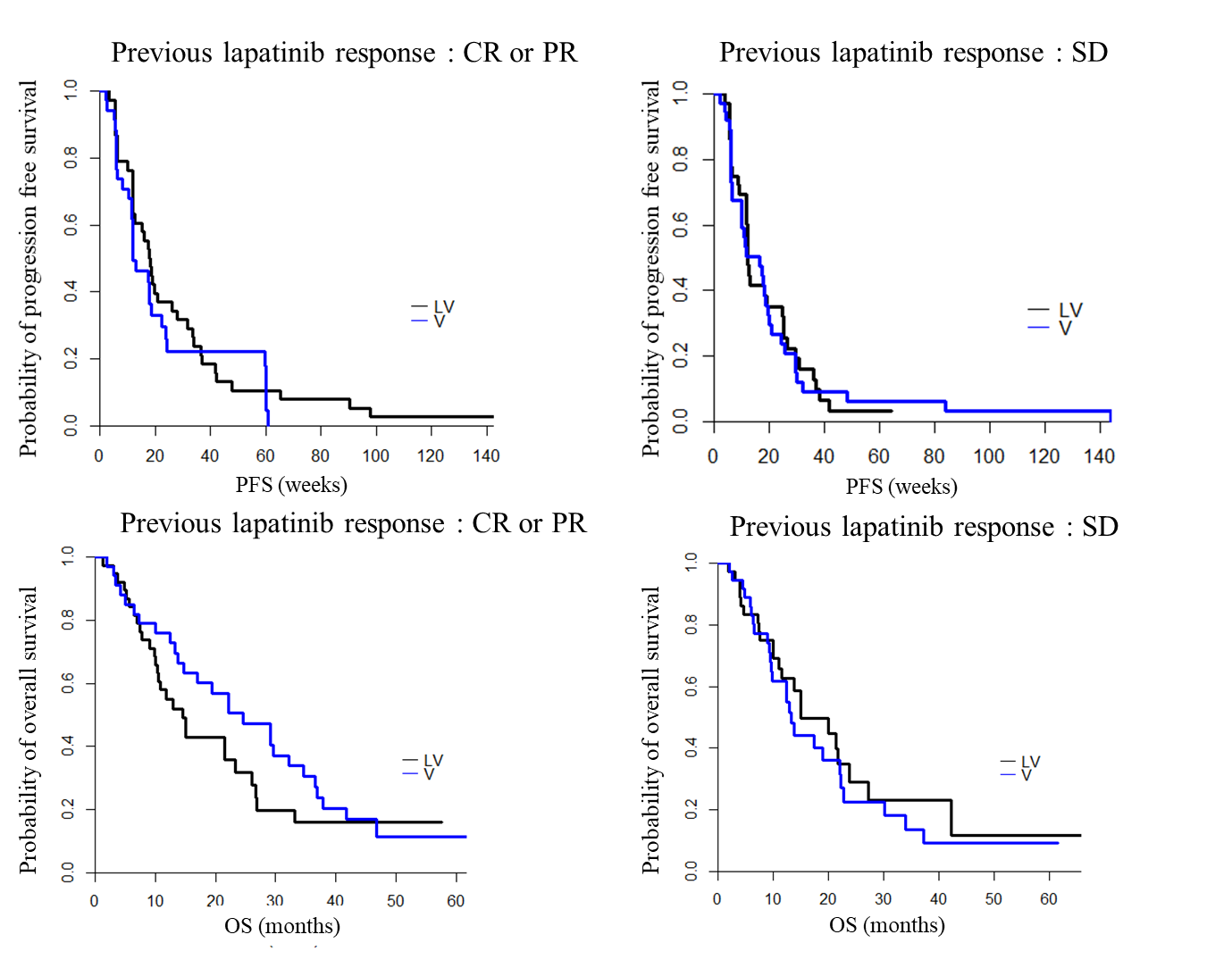


3. Supplement figure 2


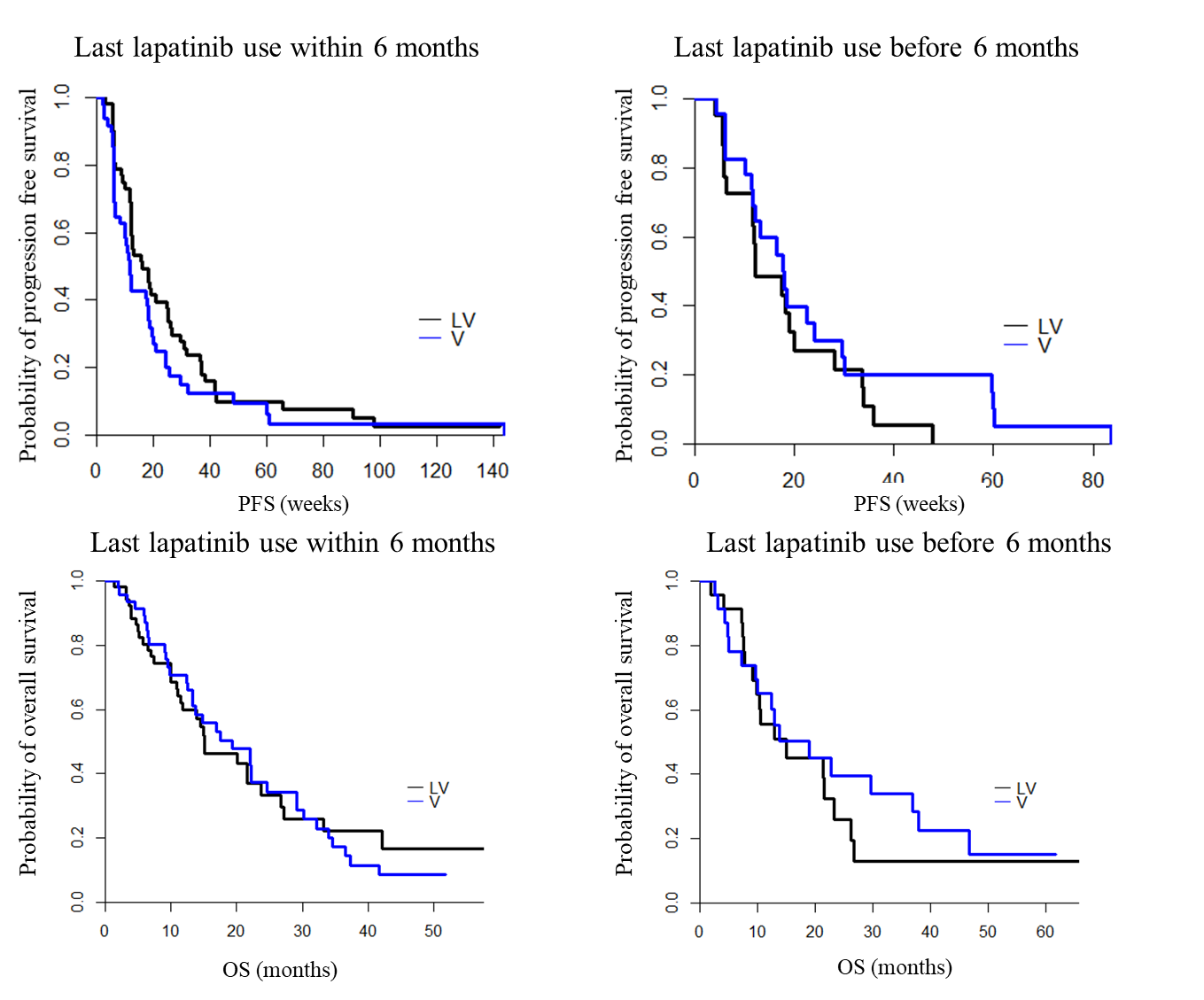


4. supplementary legends

Supplementary figure 1 : Progression free survival and overall survival according to previous lapatinib response

Supplementary figure 2 : Progression free survival and overall survival according to last lapatinib use

5. Ethical approval and consent to participate (mandatory)

- All institutional committees / approval No.

National Cancer Center Institutional Review Board / NCCCTS-11-583

Seoul National University Hospital Institutional Review Board / 1208-005420

Seoul National University Bundang Hospital Institutional Review Board : B-12-18-165-010

Samsung Medical Center Institutional Review Board : SMC2012-07-082

Yonsei University Institutional Review Board / 4-20120484

Dong-A University Institutional Review Board / 12-161

Chung-Ang University hospital Institutional Review Board / C2012-19-129(824)

Kyungpook National University hospital Institutional Review Board / KNUMC 2014-04-133

Korea University hospital Institutional Review Board / 2012AN0238
